# Altered Cingulate Cortex Functional Connectivity in Normal Aging and Mild Cognitive Impairment

**DOI:** 10.3389/fnins.2019.00857

**Published:** 2019-09-13

**Authors:** Nicoletta Cera, Roberto Esposito, Filippo Cieri, Armando Tartaro

**Affiliations:** ^1^Faculty of Psychology and Educational Science, University of Porto, Porto, Portugal; ^2^Radiology Unit, Azienda Ospedaliera Ospedali Riuniti Marche Nord, Pesaro, Italy; ^3^Department of Neurology, Cleveland Clinic Lou Ruvo Center for Brain Health, Las Vegas, NV, United States; ^4^Department of Neuroscience, Imaging and Clinical Sciences, Institute of Advanced Biomedical Technologies, D’Annunzio University of Chieti–Pescara, Chieti, Italy

**Keywords:** resting-state FC-fMRI, cingulate cortex, aging, MCI, functional connectivity, fMRI

## Abstract

**Purpose:**

Resting-state functional Magnetic Resonance Imaging studies revealed that the brain is organized into specialized networks constituted by regions that show a coherent fluctuation of spontaneous activity. Among these networks, the cingulate cortex appears to play a crucial role, particularly in the default mode network, the dorsal attention network and the salience network. In the present study, we mapped the functional connectivity (FC) pattern of different regions of the cingulate cortex: the anterior cingulate cortex, midcingulate cortex and posterior cingulate cortex/retro splenial cortex, which have been in turn divided into a total of 9 subregions. We compared FC patterns of the cingulate subregions in a sample of mild cognitive impairment patients and healthy elderly subjects.

**Methods:**

We enrolled 19 healthy elders (age range: 61–72 y.o.) and 16 Mild cognitive impairment patients (age range 64–87 y.o.). All participants had comparable levels of education (8–10 years) and were neurologically examined to exclude visual and motor impairments, major medical conditions, psychiatric or neurological disorders and consumption of psychotropic drugs. The diagnosis of mild cognitive impairment was performed according to Petersen criteria. Subjects were evaluated with Mini-Mental State Examination, Frontal Assessment Battery, and prose memory (Babcock story) tests. In addition, with functional Magnetic Resonance Imaging, we investigated resting-state network activities.

**Results:**

Healthy elderly, compared to mild cognitive impairment, showed significant increased level of FC for the ventral part of the anterior cingulate cortex in correspondence to the bilateral caudate and ventromedial prefrontal cortex. Moreover, for the midcingulate cortex the healthy elderly group showed increased levels of FC in the somatomotor region, prefrontal cortex, and superior parietal lobule. Meanwhile, the mild cognitive impairment group showed an increased level of FC for the superior frontal gyrus, frontal eye field and orbitofrontal cortex compared to the healthy elderly group.

**Conclusion:**

Our findings indicate that cognitive decline observed in mild cognitive impairment patients damages the global FC of the cingulate cortex, supporting the idea that abnormalities in resting-state activities of the cingulate cortex could be a useful additional tool in order to better understand the brain mechanisms of MCI.

## Introduction

In the last decade, a growing interest has been shown in age related abnormal changes in brain structures and functions underlying Mild cognitive impairment (MCI) and Alzheimer Disease (AD). During normal and pathological aging several cognitive, emotional and motor changes occur (Dubois and Albert, 2004; Lyketsos et al., 2011; Minhas et al., 2018). These brain age-related changes have a significant impact on individual quality of life and affect several domains (Petersen, 2004; Petersen and Negash, 2008; Metzler-Baddeley et al., 2012; Esposito et al., 2013; Cieri et al., 2017). Although the neural mechanisms underlying age-related dementias have not been well understood, several cognitive and emotional deficits can accelerate the risk of conversion to dementia. Previous studies have revealed that many brain regions are involved in emotional and cognitive age-related changes (Rombouts et al., 2005; Cieri et al., 2017).

Functional neuroimaging, in particular functional MRI (fMRI), has been extensively used to investigate the changes in brain function due to aging (Ferreira and Busatto, 2013; Gröschel et al., 2013; Brier et al., 2014) and functional changes possibly related to pre-symptomatic clinical stages of dementia, such as MCI (Fleisher et al., 2009; Greene and Killiany, 2010; Esposito et al., 2013). The functional correlation among several brain regions of the resting-state fMRI (rsfMRI) signal acquired during the resting state is considered a reliable tool to study and understand the age-related changes in several domains of cognitive functions and emotional processing. More specifically, rsfMRI studies revealed a brain organization of specialized networks constituted by regions that show a coherent fluctuation of spontaneous activity (Deco et al., 2011; Hansen et al., 2015). Indeed, several resting-state networks (RSNs) have been identified, such as the Default Mode Network (DMN), Salience Network (SN); Frontoparietal Network (FPN), primary Sensory Motor Network (SMN), Exastrastriate Visual System (EVS), and Dorsal Attention Network (DAN; Van den Heuvel et al., 2009; Van den Heuvel and Hulshoff Pol, 2010). Among these, DMN has been considered the most studied network and includes the following main nodes: the medial frontal gyrus (MedFG), posterior cingulate cortex (PCC), bilateral angular gyrus (AG) and the hippocampus (Hp; Mevel et al., 2013; Passow et al., 2015). This network is also known as the task-negative network because its regions are typically deactivated during execution of attention demanding tasks (Passow et al., 2015).

DAN, conversely to DMN - is called a task-positive network, being active during cognitive tasks which demand attention and mental control, and its function is considered fundamental during the neuroaging process. It consists of the following regions: the inferior parietal sulcus (IPS), frontal eye field (FEF), anterior cingulate cortex (ACC) and bilateral middle temporal gyrus (MidTempG; Corbetta and Shulman, 2002; Fox et al., 2005). Another RSN that received attention for its implications in the emotional processing is the SN, composed of the bilateral anterior insular cortices and dorsal cingulate cortex (DCC). During aging and in the elderly neurodegenerative conditions as MCI, these RSNs showed an altered pattern of functional connectivity (FC).

Among these networks, the cingulate cortex seems to play a key role in many cognitive, motor and emotional functions. In humans and other primates, the cingulate cortex is a thick belt of cortex encircling the corpus callosum, considered one of the most prominent features on the mesial surface of the brain (Shackman et al., 2011). fMRI studies showed the importance of this structure’s involvement among others functions with the insular cortex, the secondary somatosensory cortex, the nuclei in the tegmentum and the hypothalamus in the regulation of attentional focus by integrating external and internal stimuli, and in the expression of emotional states, thus modulating a motivational state that obtains homeostasis (Damasio et al., 2000).

Mega et al. (1996), in their proposed divisions of the limbic system, described on one hand the paleocortex part, including the amygdala, the orbitofrontal cortex, the temporal polar and anterior insula, and on the other hand the archicortical portion, including the hippocampus and ACC. The first component is responsible for the implicit integration of affects, drives and object associations; the second deals with explicit sensory processing, encoding and attentional control (Mayberg, 1994). Although divided into two sub-divisions, the paleocortex and archicortical cortex remain integrated during learning, forming a fundamentally integrated system in which the cingulate cortex seems to play a key role in emotional and affective processes, cognitive control, cognition, executing goal-directed behavior and motivation, having an important effect on social behavior (Behrens et al., 2009), psychopathology (Shin and Liberzon, 2010) and neurological disorders (Vogt, 2009).

This structure considered as a whole or considered in its subdivision into subregions seems to be involved and impaired in its functions in the pathological aging processes, playing a key role in many cognitive, motor and emotional functions, often involved in MCI. Its subregional specialization is probably related to its anatomical differentiation into cytoarchitectonically distinct subregions. According to this view, Vogt (2005) was able to isolate four principal cingulate subregions, showing an unvarying cytoarchitectonic structure and a specific pattern of connectivity, involved in different functions. The first region is the ACC, which plays an important role in affective evaluation (Allman et al., 2001), conflict monitoring, detection (Botvinick et al., 2004), response selection (Awh and Gehring, 1999) and attentional control (Posner, 1994). The second subregion is the midcingulate cortex (MCC), involved in the attention monitoring for action (Pardo et al., 1991), response selection (Corbetta et al., 1991), anticipation and working memory. The PCC appears to be more involved in the mechanisms of visuospatial orientation and body-navigation (Vogt, 2005) and in self-reflection and autobiographical memories (Spreng et al., 2009). The last subregion, the retrosplenial cingulate cortex (RSCC), plays a role in memory and visuospatial functions (Burgess et al., 2001; Keene and Bucci, 2008; Vann et al., 2009). This segregationist model attributes a single cognitive or emotional function to each single subregion.

Beckmann et al. (2009) proposed a division of the cingulate cortex into more than four regions: in a diffusion tractography study he isolated nine subregions and found that the pattern of structural connectivity, for more than one cingulate region, was overlapping.

Yu et al. (2011) tested the four-region model of the cingulate cortex, decomposing the FC pattern in seven different seeds regions, confirming the reliability of the four-region model and showing specific correlations between the several seed regions defined in the cingulate cortex and the principal RSNs.

Specifically, the ACC showed positive correlations with the DMN and anti-correlations with the visual network. The MCC was correlated with the cognitive and sensorimotor networks and anti-correlated with the visual, affective and DMN.

In a recent meta-analytic study, Torta et al. (2013) found a tripartite subdivision of the cingulate cortex during resting-state, whereas a different subdivision was observed during tasks. This tripartite subdivision involved the anterior, middle and posterior regions. This result is important for the FC during rest, and it is in line with studies on the alteration observed in the principal resting-state networks in patients affected by MCI and other neurodegenerative disorders. In fact, rs-networks, such as DMN, FPN and DAN, involve different subregions of the cingulate cortex: for instance, anterior and posterior regions of the cingulate cortex are key nodes of DMN, whereas FPN involves the midcingulate cortex (Vincent et al., 2008; Gilmore et al., 2015). Furthermore, according to Torta et al. (2013), the cingulate cortex modifies its connectivity from a resting-state to an active or “working state.” The FC pattern of the cingulate cortex appears more defined than observed during a task. Given the hypotheses from Torta and colleagues about the tripartite rs-dependent parcellation of the cingulate cortex into anterior, middle and posterior portions, we have decided to apply the same tripartition. Subsequently, according to the methods described in Yu et al. (2011), we have selected a total of 9 ROIs to study their FC pattern (and alterations) in a group of MCI patients and a group of healthy elderly. Given the subregional differentiation of the cingulate cortex and its role in several RSNs, such as DMN, DAN and SN, that suffer from aging-related cognitive and emotional changes in healthy subjects and MCI patients, we decided to compare the FC patterns of the cingulate subregions in a sample of MCI and healthy elderly subjects.

We observed specific alterations in the FC pattern in subregions of the anterior, posterior and middle cingulate. These results are globally in line with the hypothesis from Torta et al. (2013), even though we did not test our patients with cognitive or emotional tasks.

## Materials and Methods

### Participants

We conducted all procedures following Helsinki Declaration principles, and the study was approved by the Institutional and Ethics Committee of the University “G. d’Annunzio” of Chieti-Pescara. Nineteen healthy elders (age range: 61–72 y.o.; mean age: 65.2) and 16 MCI patients (age range 64–87 y.o.; mean age: 73.6) were included. All participants had comparable levels of education (8–10 years) and were neurologically examined to exclude visual and motor impairments, major medical conditions, psychiatric or neurological disorders and consumption of psychotropic drugs.

### Neuropsychological Assessment

MCI status was assigned according to the Petersen criteria (Petersen and Negash, 2008). Cognitive status of elders and MCI patients was evaluated using the following neuropsychological tests: Mini-Mental State Examination (MMSE) to evaluate the global cognitive status (26–30: normal; 25–23: MCI; <22: cognitive impairment); prose memory test (Babcock story) to evaluate prose memory (immediate recall cut-off: 5.33; delayed recall cut-off: 5.07) and the Frontal Assessment Battery (FAB; Appollonio et al., 2005) to screen for global executive functions (14–18: normal; <13: cognitive impairment). The sample was composed of 4 a-MCI single domains, 6 a-MCI multi domains and 6 non-amnestic MCI domains.

### Resting-State fMRI Acquisition

Functional and structural fMRI imaging was performed by means of a Philips Achieva 3 T Scanner (Philips Medical Systems, Best, Netherlands) using a whole-body radiofrequency coil for signal excitation and an eight-channel head coil for signal reception. During the fMRI/MRI scans, all participants were instructed to relax while fixating a white dot in the center of a gray-background screen, projected via an LCD projector and viewed via a mirror placed above the subject’s head. To minimize involuntary and voluntary head movement, a foam pad was employed. Resting-state fMRI data were collected in four runs lasting 4 min each. The small pauses between runs were used to check that patients and subjects did not fall asleep. Blood Oxygen Level Dependent data were acquired by means of T2^∗^-weighted echo planar imaging (EPI) sequences with the following parameters: TE 35 ms, matrix size 64 × 64, FOV 256 mm, in-plane voxel size 4 × 4 mm, Sensitivity Encoding (SENSE) factor 1.8 anterior-posterior, flip angle 75°, slice thickness 4 mm and no gap. Functional volumes consisted of 30 trans-axial slices, acquired with a volume TR of 1700 ms (145 volumes per run). At the end of the session, a high-resolution structural volume was acquired via a 3D fast field echo T1-weighted sequence (sagittal, matrix 256 × 256, FOV 256 mm, slice thickness 1 mm, no gap, in-plane voxel size 1 mm × 1 mm, flip angle 12°, TR 9.7 ms and TE 4 ms).

### Data Analysis: Socio-Demographic, Neuropsychological Scores, fMRI

For the fMRI/MRI data set, the following procedure was applied. The first four volumes of each functional run were discarded to allow T1 equilibration of the MR signal. MRI and fMRI data were analyzed by means of Brain Voyager QX 2.4 software (Brain Innovation, Maastricht, Netherlands). Preprocessing of functional data was performed by sequentially applying slice scan time correction, three-dimensional motion correction, and removal of linear trends from voxel time series. Motion correction was performed using a rigid body registration of EPI images to a reference image represented by the fifth volume of the first run. The root mean square (RMS) of the six realignment parameters (three translations and three rotations) was considered, in order to inspect for runs affected by excessive movement. One run for one, one run for two elders and one run for one MCI patient exceeded a root mean square (RMS) movement of 1.5 mm and were discarded from further analysis. Preprocessed functional volumes were then co-registered with the corresponding structural dataset. Both structural and functional volumes were then transformed into the Talairach space using a piecewise affine and continuous transformation. Functional volumes were re-sampled at a voxel size of 3 mm × 3 mm × 3 mm. Spatial smoothing with a Gaussian kernel of 6.0 mm full-width half-maximum was applied to functional images corresponding to two voxels in the resampled data to account for intersubjective variability while maintaining a relatively high spatial resolution. Finally, the four runs of each subject were concatenated, resulting in voxel time courses with 564 time points (423 for the few subjects that contributed with three runs). The realignment to a common reference image and the scaling to a common mean performed in the previous preprocessing steps prevented discontinuities in the concatenated timeseries.

### Definition of the Seed-ROIs

**FIGURE 1:**
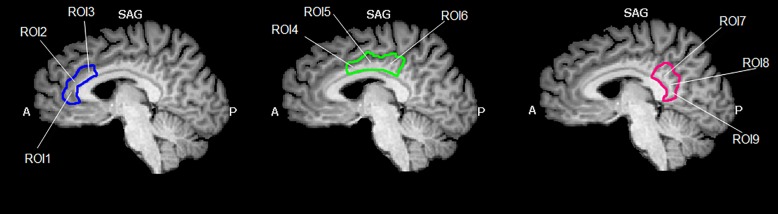
Region of interest (ROIs) used. Image shows the ROIs used for the FC analysis and their spatial location in the cingulate cortex. The cingulate cortex has been divided into three functional and anatomical parts. The blue part comprises the anterior cingulate cortex, the green part represents the set of ROIs included in the midcingulate cortex and the red one represents the posterior cingulate cortex. Images have been drawn on a Talairach atlas and in radiological convention.

The cingulate cortex has been divided into 9 regions, according to the methods described in Yu et al. (2011). Since several authors did not refer to inter-hemisphere differences, we have decided to select bilateral seed regions of interest (seed-ROIs) according to the cytoarchitectonic structural findings (Vogt and Vogt, 2003; Vogt et al., 2006). Nine bilateral regions of interest (ROIs) were determined using Talairach coordinates (Table 1 and Figure 1). Each ROI was created by means of TalCoord2VOI plug-in with a radius of 5 mm. Each ROI has been drawn in order to include the cytoarchitectonic part of the cortex and avoid the overlapping between two different ones. In particular, the seed-ROIs have been selected in order to include the anterior, middle and posterior portions of the cingulate cortex. ROI1, 2 and 3 represent the anterior and posterior portions of ACC. ROI1 is situated in correspondence with the middle BA 24 of the ACC, whereas ROI2 is positioned in correspondence with the ventral BA24 of the ACC. BA 32, the most anterior part of the ACC, is represented by the ROI3. ROI4, 5 and 6 have been selected to include the MCC. Specifically, ROI4 represents the ventral part of BA 24 in correspondence of the MCC. ROI5 includes the dorsal part of BA 24 in the MCC and the posterior part of BA 24 is represented by ROI6. ROI7, 8 and 9 include the posterior portion of the cingulate. The ventral area of PCC (BA 23) is represented by ROI7, whereas ROI8 includes the RSCC (BA 29). Finally, the dorsal PCC, also known as BA 31, is included in ROI9.

**TABLE 1:** Regions of Interest in the Cingulate cortex according the Talairach coordinates and number of voxels for MCI and Healthy Elderly.

**Cingulate cortex – seed regions**								
**Seed regions**	**Mean *x***		**Mean *y***		**Mean *z***		**Number of voxels**	
BA 24 cv	0		20		24		257	
BA24a	0		27		5		257	
BA 32	0		31		20		257	
BA a24b	0		17		30		257	
BA p24a	0		–7		40		257	
BA p24b′	0		–13		34		257	
BA 23	0		–26		29		257	
BA 29	0		–46		23		257	
BA 31	0		–34		33		257	

**Mild Cognitive Impairment**								
**Cluster**		**BA**	***X***	***Y***	***Z***	***t*-values**		***p*-values**

**Cluster 1**
Head of caudate			20	13	24	4.729		0.0000
Ventral anterior cingulate cortex		24 32	−1	22	24	18.352		0.0000
Dorsolateral prefrontal cortex		9	−34	43	18	4.749		0.0000
Inferior frontal Cortex		47	−43	28	−3	4.413		0.0000
**Cluster 2**
Entorhinal cortex		34	17	−5	−22	5.503		0.0000
Subgenual cingulate cortex		25 24 32	−1	27	0	29.986		0.0000
**Cluster 3**
Superior Parietal Lobe		7	20	−65	30	−4.056		0.0001
Anterior Cingulate Cortex		32	−1	31	18	57.775		0.0000
Head of caudate		14	19	9	4.780	0.0000
Posterior Cingulate Cortex		23	2	−53	18	4.870		0.0000
Head of caudate		−16	19	6	4.396	0.0000
Superior Parietal Lobe		7	−22	−71	27	−4.061		0.0001
**Cluster 4**
Superior Frontal Gyrus		6	44	−2	33	4.751		0.0000
Supramarginal Gyrus		40	50	−44	12	4.354		0.0000
Anterior Cingulate Cortex		32	−1	16	30	57.287		0.0000
Dorsal Posterior Cingulate Cortex		31	14	−41	36	4.287		0.0000
Middle Anterior Cingulate cortex		33	−1	19	21	−5.439		0.0000
Retrosplenial Cortex		29	−37	−38	24	−3.951		0.0001
**Cluster 5**
Ventral Anterior Cingulate Cortex		24	−1	−8	39	61.957		0.0000
Fusiform Gyrus		37	47	−53	−9	5.734		0.0000
Extra striate Cortex		18	47	−68	3	3.798		0.0002
Extra striate Cortex		19	17	−59	−21	4.737		0.0000
Dorsolateral prefrontal cortex		9	35	40	34	5.416		0.0000
Insula		13	32	22	9	3.881		0.0001
Extra striate Cortex		19	−25	−53	−25	3.376		0.0008
Putamen		−28	−5	−3	3.537	0.0005
Fusiform Gyrus/Extra striate Cortex		19 37	−49	−59	−6	5.618		0.0000
Angular Gyrus		39	−49	−65	24	−3.950		0.0001
**Cluster 6**
Dorsal Posterior Cingulate Cortex		31	17	−74	27	4.115		0.0001
Extra striate Cortex		18	8	−71	−12	4.240		0.0000
Posterior Cingulate Cortex		23	−1	−29	27	34.923		0.0000
Dorsal Posterior Cingulate Cortex		31	−10	−74	27	4.224		0.0000
**Cluster 7**
Posterior Cingulate Cortex		23	−1	−14	33	26.916		0.0000
Superior Frontal Gyrus		6	−40	−11	24	4.633		0.0000
**Cluster 8**
Middle Temporal Gyrus		21	59	−35	−3	5.226		0.0000
Supramarginal Gyrus		40	59	−41	30	−4.118		0.0000
Angular Gyrus		39	41	−59	18	11.122		0.0000
Dorsolateral prefrontal cortex		9	32	34	27	−5.816		0.0000
Insula		13	32	13	12	−4.985		0.0000
Insula/claustrum Putamen		13	32	−20	6	3.949		0.0001
Amygdala		23	−8	−12	4.612	0.0000
Anterior Prefrontal Cortex		10	2	58	15	7.954		0.0000
Extra striate Cortex		18	17	−74	−28	4.143		0.0000
Retrosplenial Cortex ventral part		30	−1	−47	21	68.122		0.0000
Superior Frontal Gyrus		6	23	−2	54	−4.504		0.0000
Extra striate Cortex		18	14	−86	−12	4.378		0.0000
Anterior Prefrontal Cortex		10	−31	37	21	−5.460		0.0000
Superior Frontal Gyrus		6	−31	7	45	6.208		0.0000
Middle Temporal Gyrus		21	−61	−23	−6	5.431		0.0000
**Cluster 9**
Inferior Temporal Gyrus		20	56	−44	−12	5.558		0.0000
Superior Temporal Gyrus		42	56	−32	15	−3.629		0.0003
Dorsal Posterior Cingulate Cortex		31	−1	−38	33	41.931		0.0000
Superior Frontal Gyrus		8	26	13	45	7.329		0.0000
Anterior Prefrontal Cortex		10	38	46	12	5.124		0.0000
Thalamus		−1	−20	6	3.586	0.0004
Superior Frontal Gyrus		6	−28	4	51	6.496		0.0000
Anterior Prefrontal Cortex		10	−19	58	12	4.444		0.0000
Para hippocampal Gyrus		35	−19	−20	−12	4.242		0.0000
Dorsal Posterior Cingulate Cortex		31	−22	−74	15	−4.177		0.0000
Dorsolateral prefrontal cortex		46	−40	46	9	3.953		0.0001

**Healthy Elderly**								

**Cluster**		**BA**	***X***	***Y***	***Z***	***t*-values**		***p*-values**

**Cluster 1**
Anterior Prefrontal Cortex		10	26	55	21	5.008		0.00
ventral anterior cingulate cortex		24	−1	22	24	16.510		0.00
Anterior Prefrontal Cortex		10	2	58	12	5.211		0.00
Anterior Prefrontal Cortex		10	−28	43	24	4.353		0.00
**Cluster 2**
Anterior Cingulate Cortex		32	−1	28	3	30.802		0.00
Extra striate Cortex		19	−16	−65	−15	−4.895		0.00
Extra striate Cortex		18	−37	−77	−18	−4.234		0.00
**Cluster 3**
Anterior Cingulate Cortex		32 9 10 24	−1	31	18	61.294		0.00
**Cluster 4**
Supramarginal Gyrus		40	59	−38	27	4.825		0.00
Superior Frontal Gyrus		6	53	−1	36	4.912		0.00
Dorsolateral prefrontal cortex		9	26	50	33	5.016		0.00
Anterior Cingulate Cortex		32	−1	16	30	58.906		0.00
Dorsal Posterior Cingulate Cortex		31	8	−29	39	5.072		0.00
Extra striate Cortex		18	-16	−80	27	4.414		0.00
Extra striate Cortex		19	-10	−47	−6	−4.609		0.00
Precentral Gyrus		4	-37	−17	27	−4.877		0.00
Insula		13	-34	22	12	4.263		0.00
Superior Frontal Gyrus		6	-58	0	21	4.787		0.00
**Cluster 5**
ventral anterior cingulate cortex		24	-1	−8	39	65.362		0.00
Dorsolateral prefrontal cortex		9	29	28	33	4.333		0.00
Extra striate Cortex		18	-4	−62	−9	4.959		0.00
Superior Parietal Lobe		5 7	-13	−53	64	4.867		0.00
Extra striate Cortex		19	-10	−44	−9	4.608		0.00
Dorsolateral prefrontal cortex		9	-31	28	30	4.919		0.00
**Cluster 6**		
Extra striate Cortex		19	29	−77	34	−5.007		0.00
Posterior Cingulate Cortex		23	-1	−29	27	32.836		0.00
Extra striate Cortex		18	-13	−71	18	5.785		0.00
Extra striate Cortex		19	-25	−80	27	−5.457		0.00
**Cluster 7**		
Posterior Cingulate Cortex		23	-1	−14	33	18.882		0.00
**Cluster 8**		
Supramarginal Gyrus		40	56	−38	33	−5.367		0.00
Superior Temporal Gyrus		22	56	−38	−6	5.418		0.00
Angular Gyrus		39	41	−65	27	10.412		0.00
Inferior Frontal Gyrus		44	47	1	18	−4.495		0.00
Dorsolateral prefrontal cortex		9	32	31	27	−5.993		0.00
Anterior Insula		13	32	19	6	−4.958		0.00
Anterior Prefrontal Cortex		10	5	58	15	8.025		0.00
Thalamus			8	−20	9	5.814		0.00
Retrosplenial Cortex		29 30	-1	−47	21	69.958		0.00
Thalamus			-7	−14	6	5.356		0.00
Dorsolateral prefrontal cortex		9	-34	34	27	−5.064		0.00
Insula		13	-34	13	9	−4.344		0.00
Angular Gyrus		39	-49	−62	15	9.489		0.00
Supramarginal Gyrus		40	-55	−38	30	−4.884		0.00
Middle Temporal Gyrus		21	-62	−8	−12	5.604		0.00
**Cluster 9**		
Superior Temporal Gyrus		42	59	−35	15	−4.390		0.00
Superior Temporal Gyrus		22	56	−47	9	−4.423		0.00
Inferior Temporal Gyrus		20	56	−47	−9	6.191		0.00
Dorsolateral prefrontal cortex		9	41	22	27	5.581		0.00
Insula		13	35	4	6	−4.344		0.00
Anterior Prefrontal Cortex		10	35	49	9	4.732		0.00
Posterior Insula		13	32	−23	0	−4.969		0.00
Superior Frontal Gyrus		8	20	19	45	7.685		0.00
Extrastriate Cortex		19	8	−50	0	5.624		0.00
Superior Frontal Gyrus		8	-25	16	45	6.828		0.00
Extrastriate Cortex		19	-37	−74	24	7.542		0.00
Anterior Prefrontal Cortex		10	-37	37	12	4.973		0.00
Extrastriate Cortex		19	-25	−77	0	−4.692		0.00
Supramarginal Gyrus		40	-34	−41	33	5.795		0.00
Dorsal Posterior Cingulate Cortex		31	-1	−38	33	41.701		0.00

### Statistical Analysis

#### Analysis of Neuropsychological Scores and Sociodemographic Data

To assess between-group differences of sociodemographic data and neuropsychological scores, a series of two-tailed *t* tests, corrected for multiple comparisons, has been applied. The Healthy Elderly group was significant younger than the MCI group (*t* = −5.11, *p* < 0.001), but the two groups showed the same levels of education (*t* = 0.70, *p* = 0.48).

The Levene test showed non-homogeneous variances for immediate recall and delayed recall (*p* < 0.01 and *p* < 0.005 respectively). Thus, a non-parametric Mann–Whitney test has been applied to assess significant differences for immediate recall (*U* = 32, *p* < 0.05) and delayed recall (*U* = 51, *p* < 0.05). The *t*-test applied for MMSE and FAB showed significant between-group differences (*t* = 9.53, *p* < 0.001 and *t* = 6.94, *p* < 0.001 respectively).

Finally, a one-way MANCOVA was performed to assess the effect of age on the testing results, showing no significant effect for the age as covariate [λ = 0.94; *F*(4,21) = 0,29; *P* = 0,88]. To assess between-group differences for the dichotomous variable of sex, a series of χ^2^ tests with Yates correction were performed. No significant differences were observed between Healthy Elderly and MCI (χ^2^= 0.6; *p* < 0.81 Yates corrected).

#### fMRI Data Analysis

Whole brain seed-based connectivity maps, related to the cingulate cortex ROIs, were created for all subjects. Then, correlations were calculated between ROI time-courses (i.e., the time-course in each of the ROI) and all the time-courses of the brain voxels. BOLD time-courses were extracted from each ROI by obtaining an average value for each voxel of the ROI modeled for each single subject. To reveal FC patterns that were consistent for the two groups (Healthy Elderly and MCI) in relation to each of the nine cingulate subregions, we proceeded in the following way: after applying Fisher’s r-to-z transformation to each correlation map, random-effect analysis was independently performed for both study groups. FC maps were computed according to the guidelines from Margulies et al. (2007). Nuisance covariates were included in the analyses to reduce effects of physiological processes such as fluctuations related to cardiac and respiratory cycles, or to motion. To this aim, we included eight additional covariates that modeled nuisance signals sampled from white matter (WM) and cerebrospinal fluid (CSF), as well as from six motion parameters (3 rotations and 3 translations as saved by the 3D motion correction). We derived WM/CSF nuisance signals averaging voxel time courses from each subject’s whole brain WM/CSF masks. These masks were generated by the segmentation process of each subject’s brain by means of brain voyager QX. After a z-normalization applied to the all seed-based predictors, the analyses, repeated with each subdivision of the cingulate cortex, were inserted in a regression model. For each seed ROI, a subject and group FC map were computed on a voxel-wise basis. For each subject, the General Linear Model (GLM) for multiple regression analysis produced 9 ROI-based t-maps and a global map based on the whole cingulate cortex. To assess between-group differences, an RFX-GLM was carried out and a mixed-design voxel-wise ANOVA, with 2 between-group levels for each seed-ROI, was performed. Statistical significance has been assessed by setting a threshold that was corrected by the False Discovery Rate (FDR) with *q* < 0.05 and *p* < 0.05. To avoid circularity effects, statistical analyses were performed following guidelines form Kriegeskorte et al. (2009). Circularity in the statistical, and specifically in the fMRI data analysis, is considered a logical fallacy occurring when the same data are used two or more times in the same analysis. This effect affects the results creating false-positive and violates the assumption of statistical independence. For this reason, we opted for anatomical selection of the ROIs and between-group comparison. However, given the significant between-group difference in the age and to study the contribution of the neuropsychological test scores to FC patterns, we performed a series of MANCOVAs on the BOLD signal extracted from the clusters resulting from the between-group comparison.

## Results

### Healthy Elderly Group FC of the Cingulate Cortex

**FIGURE 2:**
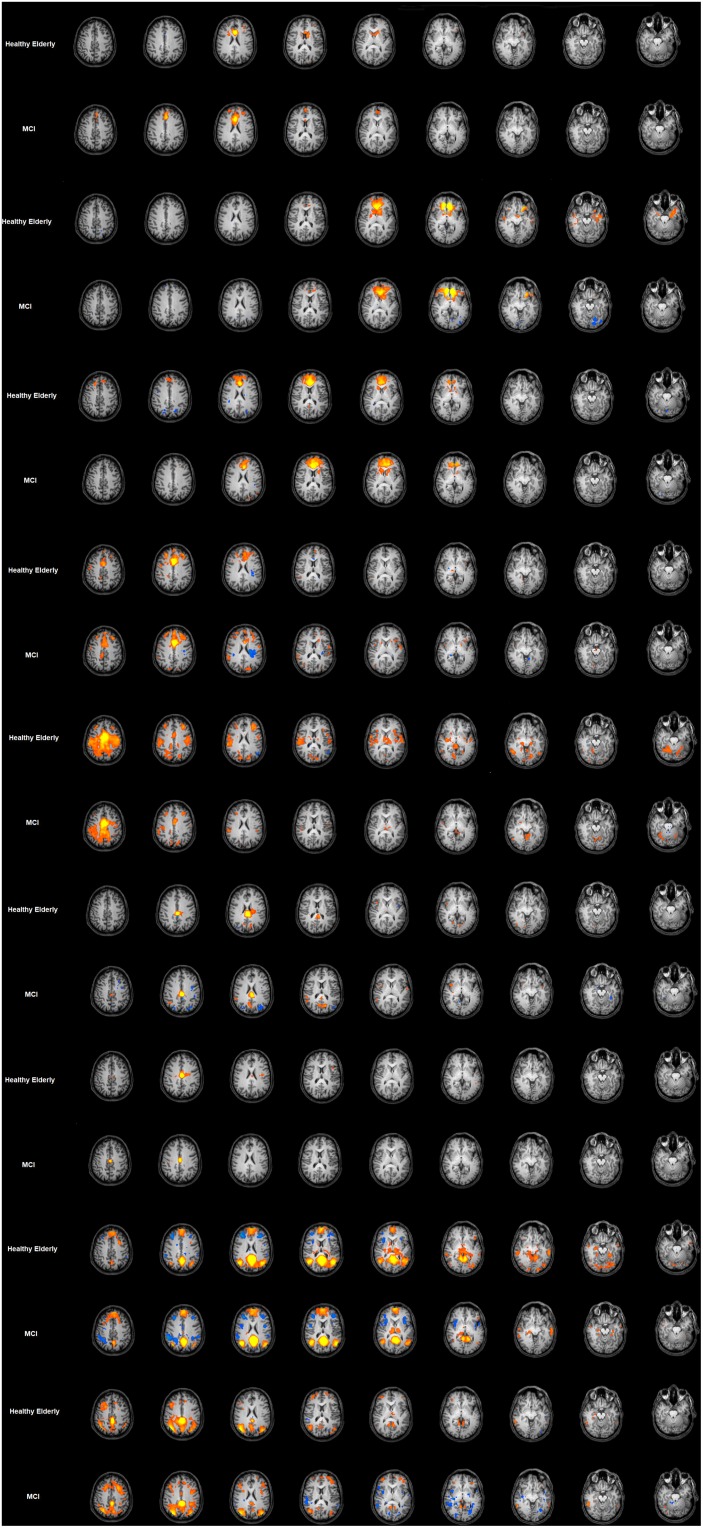
Cingulate cortex functional connectivity pattern for the elderly group and MCI. Image depicts functional connectivity patterns of the nine subregions of the cingulate cortex. Maps are superimposed on a Talairach atlas and in radiological convention with a statistical significance set at *p* < 0.05 FDR corrected. ROI FC results are listed in ascending order from the top of the figure.

The FC maps for each seed-ROI of the cingulate cortex in the healthy elderly group are shown in the Figure 2 and detailed described in Table 1. ROI1, middle BA 24 of the ACC: the FC pattern included the involvement of the bilateral anterior dorsal portion of the ACC, in particular BA 32 and the left dorsolateral prefrontal cortex (dlPFC) and the inferior frontal gyrus (IFG). Moreover, this region established a FC with the head of the caudate. ROI2, ventral BA24 of the ACC: the ventral part of BA 24 in the ACC established a FC pattern with the entorhinal cortex, in which a peak has been observed at the ventral part of BA 32 and subgenual anterior cingulate cortex (sgACC). ROI3, the most anterior part of the ACC (BA 32): BA32 showed negative FC with bilateral superior parietal lobules (SPL), whereas positive FC has been observed with the anterior part of the ACC and posterior part of the cingulate cortex, and subcortically with the head of caudate. ROI4, the ventral part of the MCC (BA 24): The pattern observed involves principally the right supramarginal gyrus and the other subregions of the cingulate cortex, like the left RSCC, MCC and ACC and the right dorsal PCC. ROI5, dorsal part of the MCC (BA 24): the dorsal regions of the MCC showed positive FC with the bilateral extrastriate cortices and the bilateral fusiform gyrus, right insula, right dlPFC and left putamen. Negative FC was observed with the left AG. ROI6, posterior part of the MCC (BA24): Established FC with extra striate cortex and posterior and dorsal PCC. ROI7, ventral area of PCC (BA 23): The ventral part of BA 23 established a FC with the left superior frontal gyrus (SFG). ROI8, retrosplenial cortex (BA 29): The RSCC showed positive FC in the right hemisphere with the MidTempG, the angular gyrus, the amygdala, the anterior prefrontal cortex, the SFG and the extra striate cortex. The same hemisphere showed negative FC with the supramarginal gyrus, the dlPFC and the insula. In the left hemisphere BA 29 showed a consistent positive FC pattern with the SFG, the MidTempG and the ventral portion of the retrosplenial cortex. Negative FC has been observed with the anterior prefrontal cortex and the SFG. ROI9- PCC: the PCC in the healthy elderly group showed positive FC in the right hemisphere with the inferior temporal gyrus, the superior frontal gyrus and the anterior prefrontal cortex. In the left hemisphere the same positive pattern has been observed with the dorsal PCC, the thalamus and the SFG. The only negative correlation has been found in correspondence with the right superior temporal gyrus.

### Cingulate Cortex FC in the MCI Group

The FC maps for each seed-ROI of the Cingulate Cortex in the MCI group are shown in Figure 2 and described in Table 1. ROI1, middle BA 24 of the ACC: The FC pattern observed in the MCI group showed the involvement of the right head of the caudate, and in the left hemisphere the ventral ACC, the dlPFC and the inferior frontal cortex. ROI2, ventral BA 24 of the ACC: The FC pattern was observed in correspondence with the right entorhinal cortex and the left sgACC. ROI3, the most anterior part of the ACC (BA 32): A positive FC pattern was found in the bilateral head of the caudate and PCC; negative FC was observed in correspondence with the bilateral SPL. ROI4, the ventral part of the MCC (BA 24): A positive FC pattern was observed in the right hemisphere in the SFG, the supramarginal gyrus and the dorsal PCC. In the left hemisphere, a positive FC pattern was observed in the MCC and retrosplenial cortex. ROI5, dorsal part of the MCC (BA 24): The FC pattern observed for the right hemisphere involved the ventral ACC, the fusiform gyrus, the extra striate cortex, the dlPFC and the insula. A positive FC pattern was also found in the left putamen, left fusiform gyrus. Meanwhile, we found negative FC in correspondence with the left angular gyrus. ROI6, posterior part of the MCC (BA24): We observed a positive FC with the bilateral dorsal PCC and the extra striate cortex in the right hemisphere. ROI7, ventral area of posterior cingulate cortex (BA 23): A positive FC was observed with the PCC and the left SFG. ROI8, retrosplenial cortex (BA 29): A positive FC pattern was observed in the right hemisphere with the MidTempG, the AG, the right insula/claustrum putamen, the amygdala, anterior PFC, extra striate cortex and ventral portion of the retrosplenial cortex. In the same hemisphere, a negative pattern has been found with supramarginal and superior prefrontal cortex and anterior insula. ROI9, PCC: A positive FC pattern was observed between the PCC and the right inferior temporal gyrus, dlPFC, anterior prefrontal cortex, SFG and extrastriate cortex. Conversely, negative correlations were observed with the right superior temporal gyrus, IFG, and anterior and posterior insula. In the left hemisphere, a positive significant FC pattern was found with the superior temporal gyrus, extrastriate cortex, anterior prefrontal cortex, suparmarginal gyrus and dorsal PCC, whereas a negative pattern was observed with the extrastriate cortex (Table 1 and Figure 2).

### Between-Group Results

**FIGURE 3:**
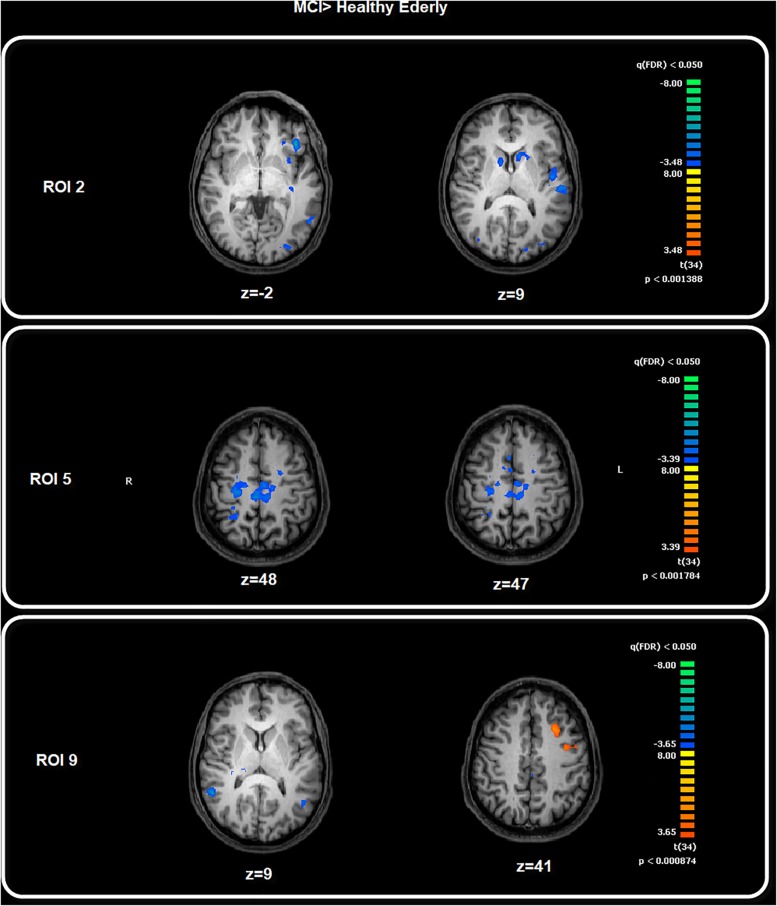
Between-group comparison: MCI > Healthy Elderly. Image depicts the significant between-group differences for the FC pattern. Maps are superimposed on a Talairach atlas and in radiological convention with a statistical significance set at *p* < 0.05 FDR corrected.

Among the FC patterns, we found only three between-group differences. Given the exploratory purpose of the present study, all the differences have been calculated on a whole brain manner, avoiding putting the focus only on specific regions involved in a given pathway. After baseline analysis, we then performed the between-group analysis for each seed ROI map, setting the significant *p* level at *p* < 0.05 FDR corrected. The comparison between healthy elderly compared with the MCI group showed a significant increased level of FC for the ROI2 in the elderly healthy group in correspondence with the bilateral caudate and left central ventromedial prefrontal cortex (vmPFC), identified with BA 10–32. Meanwhile, for ROI5 the healthy group showed increased levels of FC in correspondence with the motor cortex, S2, bilateral and right SPL. The comparison MCI with healthy elders highlighted the increased level of FC in the MCI group for ROI9 in correspondence with the middle frontal cortex, ACC and precentral sulcus (Table 2 and Figure 3). The MANCOVA was preformed to assess the effect of age on the clusters from the FC pattern of ROI2, ROI5 and ROI9 but showed no significant results (λ = 0.55, *p* = 0.11, λ = 0.49, *p* = 0.07; λ = 0.86, *p* = 0.75). No significant correlations have been observed between BOLD signal extracted for the resulting clusters of the above-mentioned ROIs and neuropsychological test scores.

**TABLE 2:** Between group comparison (MCI > Elderly) for the three seed ROIs.

		**BA**	**Hemisphere**	**Peak X**	**Peak Y**	**Peak Z**	**Number of voxels**	***t***		***p***
**ROI2: Clusters**										
Occipital Lobe		18/19	R	23	−80	37	509	−5.35		0.00
Caudate Nucleus			R	11	7	9	426	−5.07		0.00
Caudate Nucleus			L	−10	16	12	1358	−5.36		0.00
Lateral occipital cortex		18/19	L	−28	−86	15	674	−7.25		0.00
IFG		10	L	−34	22	24	1262	−4.80		0.00
Rolandic Operculum		22	L	−46	−8	6	375	−5.07		0.00
Ventral IFG		47	L	−40	28	−3	314	−6.91		0.00
ITG		21	L	−46	−53	−9	549	−5.56		0.00
STG		41	L	−55	−17	12	1569	−5.50		0.00
Lateral occipital cortex		18 19	L	−55	−59	0	1428	−6.14		0.00
Ventromedial prefrontal cortex		10 32	L	−17	38	6	624	−4.34		0.00
**ROI5: Clusters**										
IPL		41	R	38	−35	9	397	−6.22		0.00
anterior Intraparietal Sulcus		6	R	17	−17	45	2204	−6.66		0.00
Thalamus			R	20	−14	0	459	−6.70		0.00
Sensory-motor cortex		3 4	R	2	−35	60	3689	−6.92		0.00
Retrosplenial cortex/Lingual Gyrus		30	R	5	−65	9	462	−5.01		0.00
Occipital cortex		18 19	L	−16	−71	−6	2588	−7.89		0.00
Precuneus		30 29	L	−16	−62	6	639	−5.37		0.00
Caudal cingulate		32 6	L	−7	7	39	316	−5.45		0.00
Middle Frontal Gyrus		6	L	−13	−17	63	633	−5.67		0.00
Medial Frontal Gyrus		6	L	−13	1	63	330	−5.26		0.00
Anterior Insula/Putamen		13	L	−25	22	6	487	−4.78		0.00
ROI9: Clusters										
Superior Temporal Gyrus		22	R	53	−50	9	337	−6.32		0.00
Lateral Thalamus			R	32	−23	−3	684	−6.80		0.00
Posterior Cingulate		31	R	5	−32	36	586	−5.55		0.00
Superior Frontal Gyrus		8	L	−25	19	39	677	6.11		0.00
Orbitofrontral/ventrolateral prefrontal cortex		11	L	−40	34	−6	430	6.24		0.00
Frontal eye fields		6	L	−37	−5	36	488	7.32		0.00
Inferior Middle Temporal Sulcus		37	L	−49	−62	3	314	−6.85		0.00

*BA, Broadmann Areas; IPL, Inferior parietal lobe; IFG, Inferior Frontal Gyrus; ITG, Inferior Temporal Gyrus; STG, Superior Temporal Gyrus.*

## Discussion

The cingulate cortex has historically been conceived of as a multi-faceted brain structure in both cytoarchitectural and functional framework. Its high inner variability should be reflected in the processes underlying the aging and related clinical condition. In healthy young subjects it is possible to distinguish several regions based on the classification performed by Broadman in the early 1900. This classification has been used and adapted to the subsequent fMRI findings. One of the most popular classifications of the cingulate cortex, in terms of functions and anatomy, is the four-regions model theorized and studied by Vogt (2005).

We analyzed the resting-state FC patterns of the human cingulate cortex in healthy elderly and MCI individuals, for its anatomically and functionally predefined subregions (Vogt, 2005). Using a seed-based FC approach, we found that each cingulate subregion establishes connections with specific brain regions susceptible to be altered by the mechanisms underlying pathological aging decline.

Vogt’s (2005) four-region model was proposed based on integrated neurobiological assessments, and it represents a highly detailed neurobiological and anatomical model of this brain structure. The borders of the regions were defined according anatomical markers. Each region has similar cytoarchitectural structure and a common circuitry and functions. This model allows the investigation of the cingulate cortex’s functions in healthy brain, several neuropsychiatric diseases and neurodegenerative processes.

Both the ventral part of the ACC and the vmPFC are anterior parts of the DMN, and functional brain imaging studies have often shown increased activity in prefrontal brain regions in older adults. This has been proposed to reflect a compensatory shift to greater reliance on PFC, helping to maintain cognitive function (Morcom and Henson, 2018). In fact, one specific approach, the posterior-to-anterior shift in aging (PASA) theory, states that during cognitively demanding tasks the recruitment of anterior regions such as PFC contributes to maintenance of cognitive performance when posterior cortical function is impaired (Davis et al., 2008; Grady, 2012). Our results show that during resting-state the MCI group have increased FC in regions more dorsal-posterior – such as the precentral sulcus and middle frontal cortex - compared to the healthy elderly group, in which we observed a significant increased level of FC for the ventral part of ACC (ROI2) with the bilateral caudate, the vmPFC and also in the dorsal part of the MCC (ROI5), the somatomotor regions and the SPL, showing a greater ability to connect regions that are more anterior-posterior. The increased FC in regions more dorsal-posterior could be interpreted as a compensation mechanism to counteract decay affecting posterior regions primarily affected by impairment in neurodegenerative processes such as MCI and AD.

Morcom and Henson (2018) claim alternatively that activity may become less specific as people age. The authors pointed out that nevertheless that the increased activity in PFC in older adults carried less information about memory outcomes than activity in visual regions, underlying that the optimal function depends on successful brain maintenance rather than compensation.

The vmPFC is an important region connected to the ventral tegmental area (VTA), amygdala, temporal lobe, olfactory system and the dorsomedial thalamus. It sends signals to many different brain regions, including the temporal lobe, amygdala, lateral hypothalamus, hippocampal formation and cingulate cortex (Decety and Michalska, 2010). This fundamental network of connections affords to the vmPFC the ability to receive and monitor large amounts of sensory data and to affect and influence a plethora of other brain regions, particularly the amygdala. We can speculate that this increased FC with this area can support the maintenance of a better cognitive state in the healthy elderly.

In our case, FC increased in the healthy elderly group in the caudate nucleus as well as part of striatum, which coordinates multiple aspects of cognition, including motor and action planning, decision-making, motivation, reinforcement and reward perception (Ferré et al., 2010; Taylor et al., 2013; Yager et al., 2015). Striatal outputs from both the dorsal and ventral components are primarily composed of medium spiny neurons (MSNs), a type of projection neuron, which have two primary phenotypes: “indirect” MSNs that express D2-type receptors and “direct” MSNs that express D1-type receptors (Ferré et al., 2010; Yager et al., 2015). The increased FC in this area could be interpreted as good brain maintenance and an implicit monitor capable of screening the healthy elderly group regarding regions important in many aspects of cognition, motor, action planning and motivation. If damaged in their connections and in their networks, compromises typical of MCI can occur.

The caudate nucleus contributes to behavior through the excitation of correct action schemas and selection of appropriate sub-goals based on an evaluation of action-outcomes; both processes are fundamental to successful goal-directed action (Grahn et al., 2009) and our result of increased FC of ventral ACC with the caudate could be interpreted as an adaptive and successful behavior of healthy elderly in evaluation and selection tasks, an important feature for healthy cognition.

We found that cognitive decline observed in the MCI affected the global FC of the cingulate cortex and noteworthily the dorsal posterior MCC, showing a significant difference in the FC pattern in comparison to the healthy elders. The MCC can be sub-divided into two regions, which are, according to Vogt (2016), anterior and posterior. The MCI showed decreased FC in correspondence with the motor and premotor regions: the pattern of the seed ROI we defined as the “dorsal posterior MCC” was in correspondence with these bilateral motor and premotor regions. Connection with the parietal cortex plays a crucial role in modulating MCC during multisensory action monitoring, and the posterior MCC plays a primary role in the spatial reorientation of body and motor reaction to sensory and noxious stimuli (Morrison et al., 2007). In particular, the MCC processes statistical information that tracks the extent to which the outcomes of decisions meet goals when interacting with others and processes information about rewards that guide decision-making (Apps et al., 2013). This statistical processing of information seems to link this region to the “reduction of complexity” (Friston, 2010), following the approach in which the brain-mind system is perpetually committed in active inference by integrating external and internal stimuli, analyzing data from the sensorium and from external reality, trying to keep down the entropy levels and minimizing the possibility of surprise, thus modulating a motivational state that obtains homeostasis (Damasio et al., 2000). In our study, the MCC established FC connections with different portions of the parietal cortex, which as we noticed plays a crucial role in modulating the MCC during multisensory action monitoring. Recent findings suggest the existence of a frontoparietal control system consisting of flexible hubs that regulate distributed systems (e.g., visual, limbic, motor) according to current task goals (Cole et al., 2014; Cieri et al., 2017). The decreased FC in correspondence with these regions from the MCI group could represent one of the causes at the base of their cognitive impairment, being impaired in their statistical processing of information.

As proposed by Apps et al. (2013), the MCC plays an important role in processing information about the rewards others will receive and the decisions that lead to others’ rewarding outcomes.

During aging, the pattern of movements can be altered by several acquired peripheral difficulties that could affect the organization of the somatomotor brain regions. This is more evident in the MCI group. The motor cortex has been found to be involved in the programming of movements (Rizzolatti et al., 1996) and in recognition of others’ actions (Gallese et al., 2004; Fadiga et al., 2005; Goldman and Sripada, 2005; Keysers and Gazzola, 2009; Keysers et al., 2010). Perhaps in the MCI, these mechanisms implemented in the motor cortex and related to the activity of MCC should be affected by the alterations concurrent in the MCI patients. MCI subjects showed impairment in the recognition of emotional expression, particularly happy, sad and fearful facial expressions (Weiss et al., 2008). Interestingly, Weiss et al. (2008) observed the worst performance in the MCI multiple domain and not in the group of single-domain MCI patients. The functional circuit between the MCC and motor regions could play a role in the interface between the motor and emotion systems.

Recognition of an emotional expression could be affected by impairment of the memory retrieval processes, which in turn affects the planning of emotion-related body/face action/reaction. Our hypothesis is coherent with the transient impairment of motor cortex excitability, caused by transcranial magnetic stimulation (TMS), during emotion processing (Hajcak et al., 2007). Moreover, previous findings reported an indirect effect of unpleasant pictures on motor cortex excitability (Oliveri et al., 2003), which pointed out a facilitatory effect of the action on the emotions. It is plausible that the MCC and its subdivision could play a role in emotion recognition, working memory and emotion expression by means of their connections with the motor and premotor cortex, again facilitating the system in statistical information processing.

Our study highlights a new role of the cingulate cortex and in particular the MCC in the processes related to normal and pathological aging. To disentangle the role played by these subregions in mechanisms like motivation, action planning and emotions, future tasks related to fMRI and TMS studies would be helpful, possibly through a larger and more homogenous sample.

The RSCC is considered one of the key regions involved in a series of cognitive functions, such as memory and visuospatial functions (Vann et al., 2009). We observed a specific increased FC connection between retrosplenial and FEF cortices in elders.

The RSCC has been classically found to be involved during spatial navigation (Maguire, 2001) and episodic memory tasks. The FEF (Vernet et al., 2014) is responsible for saccadic movements and awareness. The increased FC between the two regions may reflect the effort to store new spatial information about the environment relevant for the spatial information recall (Hort et al., 2007; Rankin et al., 2007); moreover, it plays an important role during integration and updating of different spatial strategies. Among the functions in which it is involved, the RSCC has a key role in autobiographical memory and emotions. A recent FC study (Cheng et al., 2018) found an increased connectivity between posterior cingulate regions and lateral orbitofrontal cortex in depressed patients. It is possible that this posterior cingulate – orbitofrontal system is related to the sad memories, given that the orbitofrontal regions are involved in the non-reward system.

## Limits of the Study

Our work has some limitations that must be acknowledged. The first and most important concerns the relatively small sample size. Furthermore, another important limitation includes the heterogeneity of the MCI sample, a limitation linked to our small sample size. Increasing the sample size, homogeneity of the MCI group and improving neuropsychological evaluation in future studies would allow researchers to better address the relationship between variation in FC and cognitive performance, enhancing the significance of the present results. Moreover, cross-sectional studies could be useful to disentangle the role played by several functional brain circuits relevant in the MCI pre-AD stage, and future studies such as task-related fMRI and TMS studies would be needed to enlighten the role played by resting-state subregions in mechanisms like motivation, action planning and emotions in MCI patients.

## Conclusion

Our findings support the idea that abnormalities in resting-state activities of specific brain regions, such as the cingulate cortex, could be a useful additional tool in order to better understand the brain mechanisms of the MCI. Alterations of functional brain activity has been detected in several studies in cognitively impaired individuals. Although the modification of FC still remains unclear, it could be seen as a cerebral plastic reorganization to maintain cognitive functions (Wen et al., 2013) or instead as a neural excitotoxicity representing impending neuronal failure. The posterior-to-anterior shift in aging (PASA) theory describes the recruitment of anterior regions such as the PFC as being able to help the maintenance of cognitive performance during a cognitive task, when posterior cortical function is impaired (Davis et al., 2008; Grady, 2012). In our resting-state study the results show that the MCI group had increased FC in regions more dorsal-posterior – such as the precentral sulcus and middle frontal cortex – compared to the healthy elderly group in which we observed a significant increased level of FC for the ventral part of the ACC (ROI2) with the bilateral caudate, the vmPFC and also in the dorsal part of the MCC (ROI5), the somatomotor regions, and the SPL, showing a greater ability to connect regions more anterior-posterior. The increased FC in regions more dorsal-posterior could be interpreted as a compensation mechanism to counteract decay affecting posterior regions primarily affected by impairment in neurodegenerative processes such as MCI and Alzheimer’s. Otherwise it could implicate an altered FC mechanism, not necessarily of a compensatory nature. We also found decreased FC in correspondence with the motor and premotor regions, the “dorsal posterior MCC.” Connection with the parietal cortex plays a crucial role in modulating MCC during multisensory action monitoring. As we noted, the MCC could be linked to the statistical processing of information toward a “reduction of complexity” (Friston, 2010) through the integration of external and internal stimuli. This decreased FC in the MCI subjects could be linked to their impairment in the information processing and their consequent reduced cognitive ability. Longitudinal neuroimaging studies, homogeneity in the MCI sample, improvement of sample size and improving neuropsychological evaluation in future studies would allow researchers to better address the relationship between variation in FC and cognitive performance, enhancing the significance of the present results.

## Data Availability

The datasets for this manuscript are not publicly available. Data used in the manuscript are covered by privacy because they include sensitive patient information. Requests to access the datasets should be directed to Armando Tartaro (a.tartaro@radiol.unich.it).

## Ethics Statement

All participants gave written informed consent, and this study was approved by our local ethics committee.

## Author Contributions

NC, RE, FC, and AT conceived and designed the study. RE and FC acquired and collected the clinical data. NC analyzed the data. NC, RE, and FC wrote the manuscript. AT revised the manuscript.

## Conflict of Interest Statement

The authors declare that the research was conducted in the absence of any commercial or financial relationships that could be construed as a potential conflict of interest.
